# A Narrative Review: Epigenetic Drivers of Skin Aging and the Potential of Cellular Reprogramming to Rejuvenate the Skin

**DOI:** 10.1111/jocd.71076

**Published:** 2026-07-14

**Authors:** Ryan C. Kelm

**Affiliations:** ^1^ Clinic 5C Coeur d'Alene Idaho USA; ^2^ Wellman Center for Photomedicine Massachusetts General Hospital/Harvard Medical School Boston Massachusetts USA

**Keywords:** aesthetics, aging, cellular reprogramming, cellular senescence, epigenetics, rejuvenation, skin aging

## Abstract

**Background:**

Cellular reprogramming is an emerging technology with significant potential to prevent or reverse aging. However, its role in regenerative aesthetics as a mechanism to specifically address skin aging has not been reported.

**Objective:**

This narrative review aims to contextualize current epigenetic drivers of skin aging, highlight their central role, and provide a comprehensive exploration of the potential role of cellular reprogramming in regenerative aesthetic medicine.

**Methods:**

Literature searches were conducted to identify studies on the epigenetics of skin aging and the effects of cellular reprogramming on the skin for this narrative review. This review provides an epigenetic view of skin aging to serve as a foundation to understand findings from preclinical and animal models reporting the effects of cellular reprogramming on the skin.

**Results:**

Epigenetic changes, particularly DNA methylation drift, histone modifications, and chromatin remodeling, progressively erode cellular identity and disrupt gene expression with age. These alterations promote senescence, impair extracellular matrix homeostasis, and reduce regenerative capacity. Reprogramming methods can reset the epigenetic state, restore youthful gene expression, enhance mitochondrial function, and attenuate senescence. Preclinical studies in aged mice and human skin cells have shown that reprogramming increases epidermal and dermal thickness, accelerates wound healing, lowers inflammatory markers, and reverses epigenetic age.

**Conclusion:**

Partial cellular reprogramming represents a promising approach, ranging from prioritizing cosmetic outcomes to addressing the root causes of aging, thereby improving skin health and function. If successfully translated, partial cellular reprogramming could advance the field of regenerative aesthetics and skin rejuvenation.

## 1 Introduction

From ancient myths of ambrosia to Cleopatra's milk baths, the pursuit of youth has long shaped human culture. Yet true reversal of aging remains elusive, as most efforts focus on visible signs rather than underlying biology. Lifestyle factors and established treatments, including retinoids, photoprotection, lasers, and energy‐based devices, can enhance skin quality but mainly address downstream manifestations of skin aging.

The skin is a complex organ whose diverse cell populations progressively deteriorate with age. Intrinsic and extrinsic factors such as UV radiation, pollution, and smoking lead to epidermal thinning, impaired barrier function, delayed wound healing, dyschromia, ECM degradation, reduced elasticity, and wrinkle formation. These changes reflect deeper molecular changes of cellular identity and tissue homeostasis.

A major framework for understanding these changes is epigenetic aging. Age‐related alterations in DNA methylation, histone state, chromatin architecture, and non‐coding RNA regulation modify gene expression without changing the underlying DNA sequence. As these regulatory systems drift over time, cells lose gene control and increasingly adopt dysfunctional states. The “Information Theory of Aging” proposes that deterioration of epigenetic fidelity drives aging by disrupting the cellular information required to maintain youthful function [1].

This has made cellular reprogramming one of the most compelling areas in aging biology. By restoring a more youthful epigenetic landscape, reprogramming offers the possibility of true cellular rejuvenation rather than only phenotypic improvement. For skin aging, this has significant implications, as it suggests that interventions could restore youthful function across cell types and biological pathways rather than only improve cosmetic appearance.

Significant research into pathway‐driven and hallmark‐first approaches that prioritize skin health is already underway. Skin‐specific rejuvenating compounds have demonstrated epigenetic age reversal and restoration of youthful cellular phenotypes by targeting hallmarks of aging and longevity‐related pathways [2, 3, 4, 5]. In addition, novel skin‐specific epigenetic clocks and non‐invasive methylome profiling methods now enable more precise quantification of skin biological age and evaluation of rejuvenation interventions, outperforming older‐generation and pan‐tissue epigenetic clocks [6, 7, 8]. These milestones highlight the significant progress and growing interest in epigenetic rejuvenation, which promotes a “skingevity”‐ first strategy that focuses on the health, function, and longevity of the skin rather than cosmetic appearance alone. This narrative review explores the epigenetic drivers of skin aging and the potential of partial cellular reprogramming to prevent and reverse it.

## 2 Materials and Methods

This narrative review synthesizes current knowledge on epigenetic drivers of skin aging and the potential of partial cellular reprogramming for skin rejuvenation. The author has followed the skin aging and cellular reprogramming literature for several years, with a particular focus on studies containing any skin‐related data. PubMed searches were conducted through December 2025 using the following key terms: (“partial reprogramming” OR “transient reprogramming” OR OSKM OR OSK OR “Yamanaka factors” OR “chemical reprogramming” OR “age‐associated hallmarks”) AND (skin OR dermal OR epidermal OR fibroblast* OR keratinocyte* OR “in vivo”). Additional relevant papers were identified through citation tracking and other research efforts aimed at understanding skin aging. As this is a narrative review, systematic formal inclusion/exclusion criteria and comprehensive literature screening were not applied. Studies were selected for their mechanistic relevance to skin aging and the potential of cellular reprogramming as a mitigation strategy. The goal is to provide a broad exploration focused on the mechanistic insights and translational potential of cellular reprogramming as an emerging strategy for true regenerative aesthetics.

## 3 Results and Discussion

### 3.1 Epigenetics, Epigenetic Drift & Histone Modifications of Skin Aging

**FIGURE 1:**
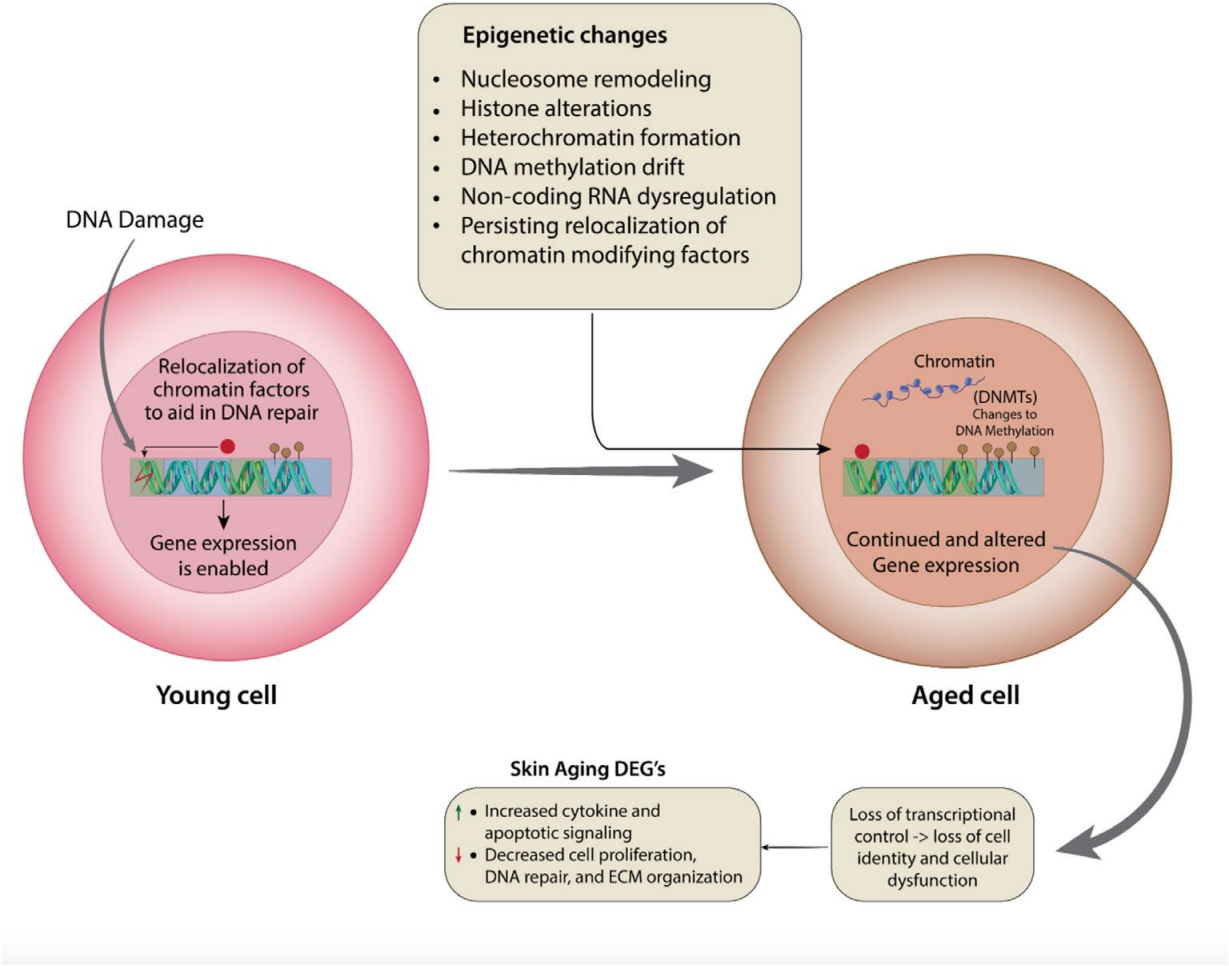
Relocalization of chromatin factors and epigenetic changes that may occur with skin aging. DEGs, differentially expressed genes; DNMTs, DNA methyltransferases.

Epigenetics encompasses the regulatory mechanisms that control how cells interpret their genome to regulate gene expression. Although most cells share the same DNA sequence, epigenetic modifications determine cell identity and function. With aging, the maintenance of epigenetic information progressively declines, leading to cellular dysfunction. Some researchers propose that this erosion of epigenetic information represents a central mechanism of aging [1]. Aging is associated with several epigenetic changes, including heterochromatin loss, histone modifications, alterations in DNA methylation patterns, dysregulation of non‐coding RNAs, and relocalization of chromatin‐modifying factors (Figure 1) [9].

DNA methylation is one of the most studied epigenetic modifications in skin aging. Age‐related changes, known as epigenetic drift, occur in both epidermal and dermal tissues [10, 11, 12]. These alterations are typically localized rather than global, affecting specific promoters, enhancers, and regulatory regions that influence gene expression [13]. In aged epidermis, hypermethylation of CpG islands and hypomethylation of heterochromatin regions have been observed. Although the overall methylome remains largely conserved, these targeted changes are sufficient to drive the aging skin phenotype, suggesting that epidermal aging results from deterioration at key regulatory sites rather than complete epigenomic collapse [13].

Methylation patterns differ between intrinsic aging and photoaging. Intrinsically aged skin shows relatively few hypermethylated sites, while photoaged epidermis tends toward hypomethylation, indicating that chronic UVR induces distinct epigenetic effects [8, 11, 14]. Clinical photoaging correlates with increasing hypomethylation [11]. More recent studies reveal that while global methylation patterns may appear similar between sun‐exposed and sun‐protected skin, hundreds of thousands of site‐specific differences exist [8].

Age‐related methylation changes also become more heterogeneous over time, starting in young adulthood and accelerating in the sixth decade [6, 8, 12]. Younger skin exhibits ordered, reproducible methylation landscapes with distinct high‐ and low‐methylation regions. In older skin, these boundaries are less distinct, reflecting epigenetic drift [6, 12, 15]. Notably, despite relatively small effect sizes, these methylation changes correlate with chronological age, allowing the development of skin aging clocks [6, 12, 16].

DNA methyltransferases (DNMTs), particularly DNMT1, help maintain methylation patterns and may contribute to skin aging. DNMT1 knockout mice exhibit premature aging, and DNMT1 expression is reduced in fibroblasts from older skin [17]. UVR also modulates DNMT1 activity [18]. For example, UVR‐induced DNMT1 upregulation can hyper‐methylate tissue inhibitor of metalloproteinase 2 (TIMP2), reducing inhibition of matrix metalloproteinase activity, thereby promoting collagen degradation [18]. Therefore, UVR contributes to dermal aging by epigenetically remodeling ECM homeostasis.

Histone modifications also play a key role in epigenetic aging by regulating chromatin accessibility and gene transcription. Histone deacetylases (HDACs) and sirtuins normally maintain chromatin structure and transcriptional balance but become dysregulated with age [19, 20, 21, 22]. Altered histone states can silence genes that should remain active or activate genes that should be repressed. Photoaging strongly associates with histone changes. Chronic UVR exposure increases histone H3 acetylation and decreases HDAC1 and SIRT1 expression compared to sun‐protected skin [23]. This increases transcription of genes involved in collagen degradation and other photoaging‐related pathways [24, 25, 26]. These findings underscore the role of histone regulation in age‐associated changes in gene expression in the skin.

### 3.2 Gene Expression and MicroRNA Regulation

Age‐related epigenetic drift influences gene expression, changing cell identity and function. Multiple transcriptomic studies have identified differentially expressed genes in aged skin, including genes involved in ECM homeostasis, metabolism, immune response, apoptosis, and cell adhesion [13, 27, 28]. Single‐cell RNA sequencing has further shown that these changes are highly cell‐type specific [27]. With age, cytokine‐mediated and apoptotic signaling pathways become upregulated, whereas epithelial proliferation and ECM organization are downregulated. Critical transcription factors that help maintain function in keratinocytes and dermal fibroblasts are also reduced, promoting senescence, impaired self‐renewal, and tissue atrophy [27]. DNA repair genes decline across several skin cell populations, further amplifying age‐related dysfunction.

However, transcript levels do not always map directly to protein levels. Studies comparing skin fibroblast transcriptome and proteome have shown only modest correlation between age‐related mRNA and protein changes [29, 30]. This highlights the importance of post‐transcriptional regulation, primarily through non‐coding RNAs such as microRNAs, during skin aging [31, 32, 33]. Although the precise interplay among DNA methylation, histone modifications, microRNAs, and gene expression in skin aging remains incompletely understood, available evidence supports a model in which relatively targeted epigenetic changes drive broader shifts in cellular phenotype.

### 3.3 Mechanistic Links Between Aging and Cellular Reprogramming

The connection between aging and cellular reprogramming is established through the reversibility of the epigenetic state. Aging is characterized by progressive dysfunction across twelve “hallmarks of aging,” including genomic instability, telomere attrition, mitochondrial dysfunction, loss of proteostasis, stem cell exhaustion, and cellular senescence [34]. Many of these hallmarks are influenced directly or indirectly by epigenetic regulation [34, 35, 36, 37, 38, 39, 40, 41, 42, 43]. Cellular reprogramming is therefore particularly significant because it can reverse several of these hallmarks by restoring youthful epigenomes.

Studies of reprogramming have shown restoration of youthful DNA methylation patterns, chromatin organization, and transcriptional programs, along with improvements in senescence markers, mitochondrial function, and other hallmarks of aging [35, 39, 42, 44, 45, 46]. These findings support the idea that epigenetic erosion is not merely a consequence of aging but may be a central driver of it. If cells retain access to a latent youthful epigenetic program, then reprogramming provides a mechanism to reactivate that state and restore function, meaning that age‐associated cellular dysfunction is not entirely fixed.

### 3.4 Cellular Reprogramming

Traditionally, aging has been considered an inevitable, unidirectional, progressive decline in physiologic function. However, cellular reprogramming challenges this. Reprogramming occurs naturally during fertilization and embryogenesis, and researchers are starting to leverage these processes to pursue aging reversal [47]. The fundamental question then becomes: Can we prevent or reverse aging, particularly skin aging?

The most widely known protocol uses the Yamanaka factors OCT4, SOX2, KLF4, and C‐MYC (OSKM), first shown to convert mouse embryonic fibroblasts into induced pluripotent stem cells (iPSCs) [38]. Since then, multiple reprogramming methods have been developed, including viral vectors such as adeno‐associated virus (AAV), mRNA‐based delivery, and chemical cocktails [39, 43, 48].

Reprogramming has two primary strategies: complete and partial. Complete reprogramming drives cells toward pluripotency, erasing somatic identity to create iPSCs, whereas partial reprogramming, also known as ‘reprogramming‐induced rejuvenation,’ restores youthful epigenetic states while preserving cell identity without full dedifferentiation [49]. This distinction is critical for therapeutic translation. Complete reprogramming carries major risks, including loss of cell identity, teratoma formation, and death in animal models when factor expression is prolonged [50, 51]. Partial reprogramming aims to capture the rejuvenating benefits of epigenetic reset while minimizing these complications.

**FIGURE 2:**
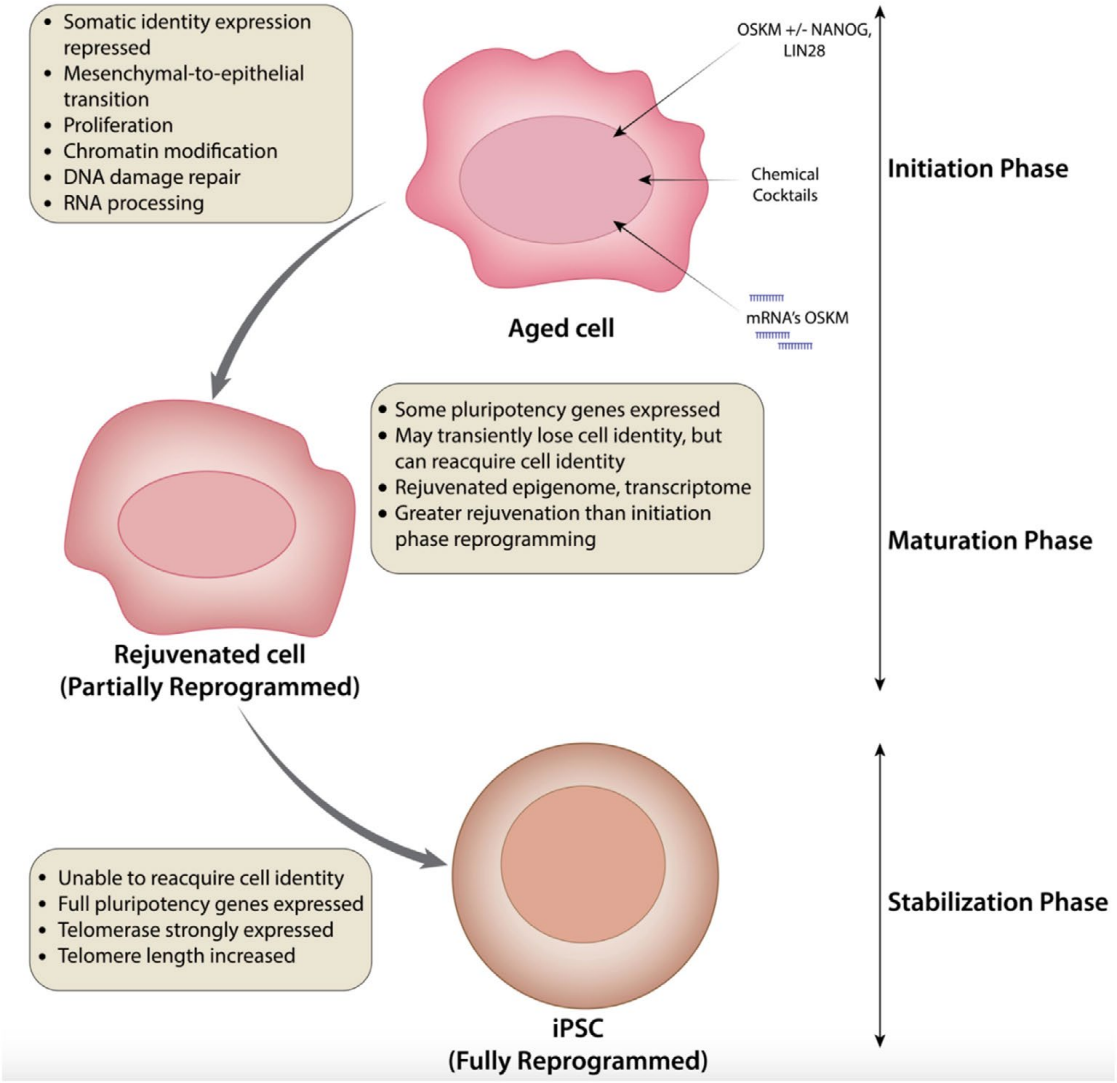
Overview of the phases of cellular reprogramming. Proposed mechanisms and events, by phase, based largely on preclinical data.

Reprogramming proceeds through initiation, maturation, and stabilization phases, and the biological outcome depends heavily on timing, dosing, tissue context, and the model's age (Figure 2) [52, 53, 54]. For example, functional rejuvenation was more significant in damaged, diseased fibroblasts than in wild‐type mouse tail‐tip fibroblasts [55]. In addition, healthy models may be less susceptible to reprogramming, the effects of OSKM may be cell‐type specific, and an extended duration of short‐term, cyclic reprogramming may lead to more significant rejuvenation [42, 56, 57].

The initiation phase appears to be especially relevant to rejuvenation. Early in reprogramming, cells exhibit enhanced proliferation, chromatin remodeling, and DNA repair, and reduced epigenetic age in mouse embryonic fibroblasts [55, 58, 59, 60]. Later stages may further deepen rejuvenation. One maturation‐phase transient‐reprogramming strategy showed that human dermal fibroblasts could transiently lose and then reacquire their identity, resulting in rejuvenation of the transcriptome, epigenome, and DNA methylation age by approximately 20 to 40 years, while increasing type I and IV collagen toward youthful levels [61].

Overall, partial reprogramming has been shown to reverse aging phenotypes and increase healthspan and lifespan in mice [35, 39, 42, 57, 61, 62]. Short‐ and long‐term cyclic and continuous OSKM expression, within the partial reprogramming window, rejuvenates the epigenome, decreasing the epigenetic age of keratinocytes and fibroblasts, attenuating senescence, improving mitochondrial function, reducing DNA damage, and restoring youthful gene expression [35, 42, 43, 62]. Specifically, these effects occur early, typically within the first 7–17 days of OKSM expression, well before full pluripotency is achieved with complete reprogramming, which generally requires 30–50 days in standard iPSC protocols [38, 61, 63, 64, 65]. These findings suggest that rejuvenating benefits can be achieved before cells reach full pluripotency, providing a therapeutic window in which epigenetic age is reduced while cell identity is preserved.

### 3.5 Effects of Cellular Reprogramming on Skin Cells

The field of reprogramming‐induced rejuvenation is rapidly growing, and evidence specifically addressing the skin has already shown increasingly compelling results (Table 1). Dermal fibroblasts are among the most commonly used models in in vitro studies of partial cellular reprogramming. They were among the first cell types in which partial reprogramming was shown to induce a steady decline in epigenetic age before loss of somatic identity [63]. In addition, in vivo animal models further support the effects of reprogramming‐induced rejuvenation on the skin. However, all evidence remains preclinical.

**TABLE 1:** Published reports of cutaneous cellular reprogramming.

Author (year)	Evidence quality/type	Reprogramming method	Model tissue	Duration	Frequency	Results
Ocampo et al. (2016) [35]	Peer‐reviewed, preclinical, in vivo	Doxycycline‐inducible OSKM	Mice	6 weeks	2 days on/5 days off	Increased epidermal thickness Increased dermal thickness Restored skin proliferation rate
Olova et al. (2019) [63]	Peer‐reviewed, preclinical, in vitro	OKSM transfection	Human dermal fibroblasts	49 days	Continuous	Steady decline of epigenetic age by 3.8 years per day of OSKM expression from day 3 to 20 Majority of fibroblast marker genes remained highly expressed until day 15 By day 35, all fibroblast clusters loss somatic identity
Alle et al. (2022) [57]	Peer‐reviewed, preclinical, in vivo	Doxycycline‐inducible OSKM	Mice (2 months old)	2.5 weeks	Continuous	Increased epidermal and dermal tissue thickness ~40% Increased subcutaneous tissue thickness ~120% Reversed age‐related methylation status of skin
Browder et al. (2022) [42]	Peer‐reviewed, preclinical, in vivo	Doxycycline‐inducible OSKM	Mice *Long‐term* 15 and 12 month old *Short‐term* 25 month old	*Long‐term* 7‐ and 10‐months, respectively *Short‐term* 1‐month	2 days on/5 days off	*Long‐term* Significant decrease in skin epigenetic age Larger impact on DEGs toward youth Increased proliferative capacity of skin cells Increased regenerative capacity and reduced fibrosis in wounds *Short‐term* No change in epigenetic age DEGs more similar to younger levels
Gill et al. (2022) [61]	Peer‐reviewed, preclinical, in vitro	Doxycycline‐inducible OSKM, plasmids/lentivirus	Human fibroblasts	10–17 days (13 days optimal)	—	Increased collagen I, collagen IV gene expression and protein to youthful levels Improved migratory capacity Increased age‐related decline in epigenetic marker H3K9me3 Significant reduction of DNA methylation age ~30 years Restored youthful transcriptome
Schoenfeldt et al. (2025) [66]	Peer‐reviewed, preclinical, in vitro	Chemical cocktails	Aged human fibroblasts	6 days	—	Decreased DNA damage marker γH2AX Restored youthful epigenetic marks Decreased Senescence‐associated cell‐cycle gene expression Upregulated genes associated with cellular reprogramming, self‐renewal, and stemness
Sarkar et al. (2020) [39], Monteleon et al. (2023) [67]	Peer‐reviewed, preclinical (Sarkar); conference abstract (Monteleon); in vitro	mRNA‐OSKM + LIN28, NANOG	Aged human fibroblasts; skin explant cultures	4 days	—	Gene expression resembled youthful profiles Increased ECM and collagen genes Decreased MMPs and oxidative stress genes DNA methylation age reversal Restored youthful epigenetic markers Increased mitochondrial potential, and SIRT1, reduced mitochondrial ROS Decreased senescence related p16 expression Increased cell proliferation
Macip et al. (2024) [62]	Peer‐reviewed, preclinical, in vitro and in vivo	AAV, doxycycline‐inducible OSK Plasmids/lentivirus	Human keratinocytes 124‐week‐old male mice	Till end of life	1 week on/1 week off	Human keratinocytes: Epigenetic age reversal by DNA methylation age Mice: Increased median remaining lifespan by 109% relative to wild‐type mice

Abbreviations: AAV, Adeno‐associated virus; DEGs, differentially expressed genes; ECM, extracellular matrix; MMPs, matrix metalloproteinases.

In mice, doxycycline‐induced OSKM expression increased epidermal and dermal thickness and restored proliferative activity in the skin [35]. Longer‐term partial reprogramming in naturally aged wild‐type mice reduced biological age in the skin, lowered markers of senescence and inflammation, increased epidermal proliferation, increased skin thickness, and improved wound healing [42]. Interestingly, skin appeared more responsive than several other tissues in methylation and transcriptomic analyses, suggesting that skin may be particularly susceptible to rejuvenation through reprogramming.

Another study delivered OSK to 124‐week‐old wild‐type mice and to human keratinocytes and found a 109% increase in remaining median lifespan, improved healthspan, and a significant reversal of epigenetic age in human keratinocytes [62]. The human age comparable to that of 124‐week‐old mice is around 77 years, suggesting that the effects of cellular reprogramming may still be beneficial as a late‐life therapeutic.

A key translational question is durability. Some work suggests that reprogramming benefits diminish after withdrawal of the inducing factor, implying that continued maintenance strategies may be necessary [35]. However, another study showed durable improvement. In one model, a single 2.5‐week OSKM burst in young mice produced sustained skin benefits and a rejuvenated DNA methylation profile that persisted months later, delaying age‐related deterioration. Epidermal and dermal thickness increased by 40%, while the superficial subcutaneous fat increased by 120%, and age‐related methylation sites were restored [57]. Interestingly, the methylation sites altered immediately after reprogramming were not identical to those observed later, suggesting that reprogramming may initiate a longer‐term propagation of youthful epigenetic organization during subsequent aging.

Non‐integrating approaches are also being explored to improve safety. mRNA‐based delivery of reprogramming factors, also including LIN28 and NANOG, has shown prompt, cell‐type‐specific rejuvenation of epigenetic and transcriptional signatures before loss of identity genes [39, 67]. In human skin fibroblasts, this has increased SIRT1 expression, increased mitochondrial membrane potential, reduced mitochondrial reactive oxygen species, enhanced ECM protein deposition, increased collagen‐related gene expression, reduced MMP expression, decreased oxidative stress signaling, increased heterochromatin‐associated markers, reduced p16, and improved proliferation [39, 67]. The restoration of fibroblasts through reprogramming increases youthful function and reestablishes youthful gene expression via transcriptomic changes primarily observed in aged skin fibroblasts [27, 67]. Chemical reprogramming offers an alternative by avoiding the introduction of DNA or mRNA altogether [43]. In human dermal fibroblasts, combinations of small molecules have improved several hallmarks of aging, including DNA damage, senescence‐associated gene expression, and heterochromatin state, though the effects appear less robust than those achieved with OSKM‐based approaches [66]. Although these data are less well established than those for OSKM‐based reprogramming, they suggest that partial activation of rejuvenation‐associated pathways may eventually be achievable through less invasive approaches.

### 3.6 Advantages of Cellular Reprogramming for Skin Aging

While many interventions aim to mitigate visible signs of skin aging, few directly target the underlying cellular mechanisms driving the process. Topical agents may improve barrier function, cellular turnover, or collagen production, while lasers and energy‐based devices enhance texture, dyspigmentation, and dermal remodeling through controlled injury. These modalities provide meaningful cosmetic benefits but generally do not reset the cell's fundamental aging state.

Cellular reprogramming is conceptually different because it addresses root epigenetic causes. By restoring a youthful epigenetic state, it simultaneously improves mitochondrial function, proteostasis, DNA repair, stress resilience, and reduces senescence burden. This multi‐hallmark effect distinguishes it from therapies focused on a single downstream mechanism. For example, senolytics or senomorphics may improve a single major hallmark of aging, whereas reprogramming appears capable of affecting several hallmarks simultaneously, providing additional lifespan advantages [40].

Depending on timing and protocol, cellular reprogramming may serve as either treatment or prevention. In older cells with significant epigenetic noise, it acts as a treatment by resetting the epigenome and reversing age‐associated phenotypes. In younger cells, carefully timed reprogramming may function as maintenance, preserving youthful function and delaying decline. For example, intradermal AAV‐OSK administered prior to wounding improved subsequent wound healing in mice [40]. Future clinical strategies may therefore include both therapeutic and preventative applications.

### 3.7 Limitations and Future Directions

Partial cellular reprogramming is approaching clinical translation. As of early 2026, the FDA has cleared the IND for the first Phase 1 human trial of partial cellular reprogramming (Life Biosciences' ER‐100, OSK‐based gene therapy for optic neuropathies). While not specific for a dermatologic or cosmetic indication, it highlights progress in the field toward clinical use. Nevertheless, key challenges remain in safety, delivery, efficiency, durability, and tissue specificity.

The risk of tumorigenesis and unintended dedifferentiation is a major concern, especially for in vivo use. A particular concern in aesthetic patients is the high burden of photodamage and field cancerization, in which UV‐induced p53 mutations create keratinocyte clones that are predisposed toward skin cancer development. Because normal p53 restrains reprogramming, whereas p53‐mutant cells are more easily reprogrammed, it is unknown whether partial reprogramming could preferentially activate or expand these clones [68, 69]. In addition, partial reprogramming led to dedifferentiation of melanoma cells, increasing their invasiveness, and premature termination or prolonged OSKM reprogramming, in vivo, can drive dysplasia and tumors [50, 51, 70]. However, multiple recent in vivo mouse models have shown no increase in tumorigenesis with partial reprogramming, and the removal of c‐MYC reduces oncogenic risk [40, 71, 72, 73]. Furthermore, in a lung adenocarcinoma model, transient OSKM expression was anti‐tumorigenic [74]. Nevertheless, skin‐specific studies on photodamaged skin with premalignant and malignant lesions will be essential before this can enter into clinical trials. It will be important to determine whether partial cellular reprogramming attenuates or amplifies the premalignant/malignant phenotype. Protocols will require long‐term cancer surveillance and must be optimized to achieve safe partial reprogramming without progression to pluripotency, immune responses to viral vectors, and off‐target effects. Skin‐specific reprogramming strategies are critical, as systemic activation of reprogramming factors risks confounding or harmful off‐target effects. Local delivery systems and topical induction may provide safer translational pathways. For example, topical doxycycline activated OSKM in wound models, thereby reducing fibrosis and scar formation. These findings suggest that localized cutaneous activation of OSKM may be feasible [75].

Durability is still uncertain and remains one of the biggest open questions. It is unclear how long benefits persist, whether repeat treatments will be required, and how reprogrammed cells continue to age. Responses also vary by tissue type, disease state, chronological age, and the duration or frequency of exposure to the factor. Diseased or damaged cells often respond more robustly than healthy cells, and skin cells may vary in susceptibility and durability of effect.

Another limitation is the extrapolation of these results to the potential for clinical skin rejuvenation. Although preclinical studies in mice and human skin cells have shown molecular, cellular, and histologic improvements such as reversed epigenetic age, reduced senescence, and increased collagen and epidermal/dermal thickness, these results should not be directly equated to clinical skin rejuvenation. Preclinical work must shift toward models that more closely resemble human skin biology in vivo, and the primary outcomes should be clinically relevant, including wrinkles, dyspigmentation, barrier function, scar outcomes, and elasticity. These molecular changes may then correlate with clinical improvements.

Future studies should assess the impact of reprogramming beyond DNA methylation, including histone modifications, non‐coding RNA regulation, and broader changes in gene expression associated with skin aging, while also examining non‐cellular effects on ECM reorganization and other macromolecules. Important skin‐specific endpoints include: histologic measures (epidermal and dermal thickness; collagen—quantify and qualify, subtype ratios, and organization pattern; elastic network architecture, presence of immature fibers (elaunin, oxytalan), degree of elastotic debris), cellular measures (proliferation, migration, differentiation, morphology), molecular markers (multi‐omic data beyond epigenetic clocks, senescence‐related biomarkers (p16, p21, SA‐ß‐gal, senescence‐associated secretory phenotype), MMP expression), functional evaluation metrics (transepidermal water loss, cutometer‐based elasticity, wound healing rate, tensile scar strength), clinical outcome measures (pigment, texture, rhytides), and long‐term cancer surveillance.

Reprogramming will unlikely be a monotherapeutic panacea and will likely need to be combined with other controlled wounding modalities to elicit more robust tissue rejuvenation [76]. Reprogramming coupled with lasers and energy‐based devices may lead to broad synergistic effects on cellular and non‐cellular tissue rejuvenation. Ultimately, human clinical trials focused on aesthetic and regenerative endpoints will be essential to confirm whether epigenetic rejuvenation delivers meaningful improvements in skin quality, wound healing, resilience, and appearance.

## 4 Conclusion

**FIGURE 3:**
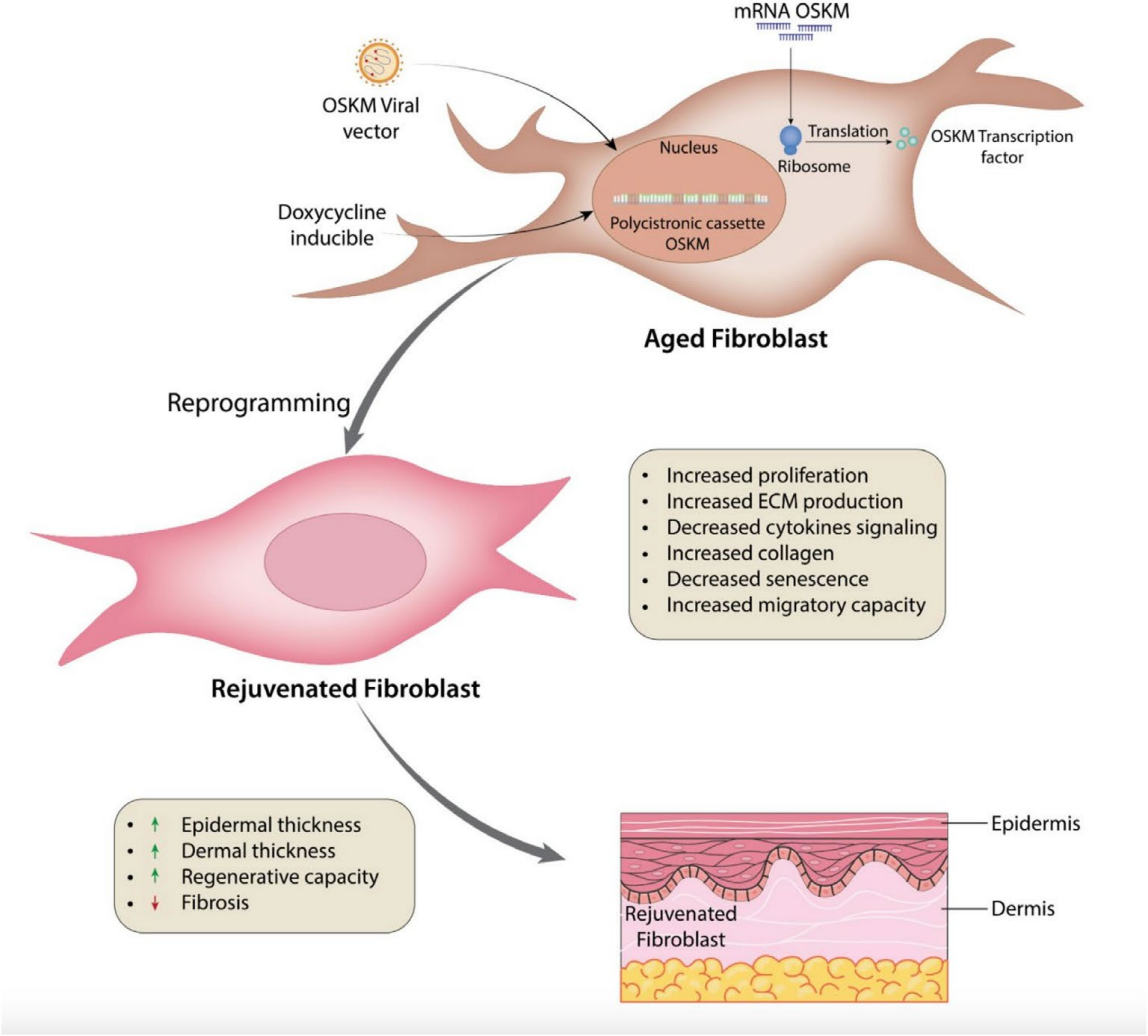
Overview of the proposed mechanisms and effects of cellular reprogramming on the skin based on preclinical data.

Skin aging is increasingly understood as a process shaped by epigenetic drift, altered gene regulation, and progressive loss of cellular identity and function. These changes affect multiple skin cell populations and contribute to the structural, functional, and aesthetic decline associated with aging. Cellular reprogramming is disruptive because it directly challenges the assumption that these changes are irreversible. Its ability to reverse age‐related epigenetic modifications and restore youthful cellular function is a significant breakthrough. By resetting epigenetic state, reprogramming has been shown to improve senescence, mitochondrial dysfunction, DNA damage, gene expression, proliferative capacity, and tissue‐level features of aging in animal models (Figure 3).

Although many challenges remain, the field has already demonstrated that aged skin cells can be shifted toward a more youthful state. This makes cellular reprogramming fundamentally different from conventional anti‐aging interventions that primarily target aging phenotypes. Rather than merely improving the appearance of aged skin, reprogramming may eventually allow restoration of youthful cellular function itself. If successfully translated, it could advance the field of regenerative aesthetics and skin rejuvenation.

## Funding

The author has nothing to report.

## Ethics Statement

The author has nothing to report.

## Conflicts of Interest

The author declares no conflicts of interest.

## Data Availability

The author has nothing to report.
